# Clinical status and assessment of caries on first permanent molars in a group of 6‐ to 13‐year‐old Tunisian school children

**DOI:** 10.1002/cre2.676

**Published:** 2022-10-20

**Authors:** Farah Chouchene, Fatma Masmoudi, Ahlem Baaziz, Fethi Maatouk, Hichem Ghedira

**Affiliations:** ^1^ Pediatric and Preventive Dentistry Department Faculty of Dental Medicine of Monastir Monastir Tunisia; ^2^ Laboratory of Biological, Clinical and Dento‐Facial Approach University of Monastir Monastir Tunisia

**Keywords:** dental caries, enamel defects, first permanent molar, Gingivits

## Abstract

**Aim:**

The present survey aimed to highlight the clinical status of first permanent molars (FPMs) among a group of children aged between 6 and 13 years in public primary schools in Tunisia and to determine the association between FPMs' dental caries, gingival bleeding, malocclusion, dental fluorosis, and enamel defects.

**Materials and Methods:**

The survey involved a cross‐sectional study based on a dental examination conducted in public primary schools in Monastir Tunisia. A dental caries assessment was performed on FPMs using the International Caries Detection and Assessment System classification; the number of carious lesions in permanent and temporary dentition was established using the decayed/missing/filled teeth (DMFT) index. The Gingival Index and the Dental Aesthetic Index were used to determine the occurrence of gingival bleeding and malocclusions. Dean's index and the modified Development Defects of Enamel index were used to define the enamel defects. The *χ* ^2^ test was used to assess the difference between more than two groups, and the level of statistical significance was set at .05%.

**Results:**

A total of 545 children and 2080 FPMs were examined. The prevalence of dental caries in FPMs was recorded to be 35.8%. The overall mean DMFT index of the study population was 1.62 and the mean DMFT index was 1.41. The proportion of carious FPMs increased significantly with the age of the children (*p* < .05). The mandibular first permanent molar presented higher caries prevalence than its maxillary counterpart (*p* < .05). The presence of surfaces affected by molar incisor hypomineralization was recorded in 4.3% and the presence of surfaces affected by fluorosis was recorded in 4.6% of FPMs. No association was reported between FPMs' dental caries, gingival bleeding, malocclusion, and enamel defects (*p* > .05).

**Conclusion:**

Although the prevalence of caries in FPMs was considered moderate, health promotion programs should be implemented on a large scale to decrease the prevalence of dental caries among school children.

## 1 INTRODUCTION

First permanent molars (FPMs), which usually emerge at the age of 6 years, are considered the most important permanent teeth on the dental arch because of their numerous roles in the development and maintenance of dental occlusion (Varshney et al., 2018).

FPMs, representing ∼50% of the masticatory efficiency, serve not only as a guide for the emergence of the adjacent molars but also govern lifelong mastication and are responsible for the establishment of the second physiological phase of occlusion; they are also considered to play a key role in angle occlusion (Griffin et al., 2012; Pontigo‐Loyola et al., 2020; Sánchez‐Pérez et al., 2019).

Due to their anatomical structure, early emergence in the mouth, and the circumstances in which they appear in the mouth, without exfoliating primary teeth, FPMs require special attention as they are very prone to caries and cavities (Al‐Samadani & Ahmad, 2012; Pontigo‐Loyola et al., 2020). They are thus exposed to risk factors and progressive dental caries, which often result in their destruction (Pontigo‐Loyola et al., 2020; Sánchez‐Pérez et al., 2019).

Besides, FPMs, the mineralization period of which coincides with childhood diseases, can emerge with structural abnormalities. Molar incisor hypomineralization (MIH) is considered to be the most common defect observed on FPMs in children and adolescents but other uncommon abnormalities can also affect these teeth such as shape, size, and/or eruption abnormalities; therefore, in many cases, early diagnosis of these types of lesions can allow avoidance of complications and the systematic use of endodontic and prosthetic treatments and, in some cases, even tooth extraction (Hamza et al., 2019). Indeed, the lack of proper oral hygiene among children, the increased risk of caries and structural abnormalities of FPMs, and the limited knowledge of parents of the correct cleaning methods can result in the early loss of FPMs (Saber et al., 2018).

Thus, it is important to recognize the frequency and severity of caries affecting FPMs to establish programs to prevent their decay. There is very limited research on the prevalence of caries in FPMs in Tunisia; for this reason, the present study was designed with the aim of exploring the present situation among primary school children aged between 6 and 13 years in a Tunisian community.

## 2 MATERIALS AND METHODS

### 2.1 Study design and setting

The present survey was designed as a cross‐sectional study, conducted in public primary schools located in Monastir, Tunisia, from April to June 2019.

### 2.2 Study participants

Primary school children, aged 6–13 years of grades 1–6 (grade is 1‐year education course), living in Monastir, Tunisia, were included in the present survey.

The inclusion criteria for participation in the present study were as follows: willingness to participate and the presence of at least one FPM.

The sample size was estimated using the following assumptions: *α* error = 4%, study power = 95%, and estimated caries prevalence of 65% (Zouaidi et al., 2012). The minimum required sample size was calculated to be 540. A three‐stage stratified cluster random sampling technique was applied to select the study population.

### 2.3 Data collection

A questionnaire was designed to record the personnel profile of the children.

Using the International Caries Detection and Assessment System, a dental caries assessment was performed on FPMs (teeth 16, 26, 36, and 46).

The number of carious lesions in permanent and temporary dentition was established by the sum of decayed/missing/filled teeth (the DMFT index). Gingival bleeding was determined according to the Gingival Index (Gingival and periodontal indices-periobasics.com Clinical Periodontology, 2020). Dental fluorosis was determined based on Dean's index, and MIH defects were assessed according to a modified Development Defects of Enamel index (Clarkson & O'Mullane, 1989).

Malocclusion was reported using the Dental Aesthetic Index. The classification of normal occlusion was performed according to the following criteria: class I relationship of FPMs, canines in normal relationship; absence of anterior or posterior crossbite, vertical and horizontal overjet not surpassing 2 mm, presence or absence of inter‐incisor diastema, absence of crowding, absence of tooth anomalies in shape and number, and absence of tooth rotation (Cardoso et al., 2011).

All children received oral hygiene instructions and an oral hygiene hamper that consisted of toothpaste and a toothbrush. After the children had brushed their teeth under a dentist's supervision, oral examinations were perfomed by two previously trained pediatric dentists with an acceptable inter‐examiner agreement (*κ* ≥ .06), using a periodontal probe and a mouth mirror under natural light with the examiner standing and the child seated. The findings were recorded on an individual clinical chart for each child.

### 2.4 Statistical analysis

Statistical analyses were performed using the IBM Statistical Package for Social Sciences version 22.0 for Windows. Descriptive statistics and frequencies were generated. The *χ* ^2^ test was used to assess the difference in the means between more than two groups. A comparison was made between gender, school grades reflecting the age groups, tooth brushing frequency, and between maxillary versus mandibular FPMs. The level of statistical significance was set at .05%. The inter‐examiner agreement was analyzed using Kappa statistics, and a high degree of agreement was observed between the two examiners (*κ* ≥ .06).

### 2.5 Ethical approval

The study was approved by the research committee of the Faculty of Dental Medicine of Monastir, and visitation permissions for the selected schools were obtained from the Regional Delegation of the Ministry of Family and Child, Monastir, Tunisia.

Before the survey, informed consent forms that were completed by the children's parents were obtained.

Children who required dental treatment and/or interceptive orthodontic treatment were referred to the Pediatric Dentistry Department at the clinic of the Dental Medicine of Monastir.

## 3 RESULTS

A total of 545 children aged between 6 and 13 years with a mean age of 9.62 ± 0.77 years were enrolled in this cross‐sectional survey, and a total of 2080 FPMs were examined.

In all, in 64.2% of children (350 children), all FPMs were intact and in the remaining 35.8% of children (195 children), one or more FPMs had caries. The overall mean DMFT index of the study population was 1.62, and the mean DMFT index was 1.41.

The characteristics of the children who participated in our survey are summarized in Table 1.

**Table 1:** Description of the characteristics of the participating children

	*N*	%
Gender		
Boys	280	51.4
Girls	265	48.6
Age group (years)		
6–7	71	13
7–8	26	4.8
8–9	44	8.1
9–10	143	26.2
10–11	100	18.3
11–12	148	27.2
>12	13	2.4
Tooth brushing frequency		
Less than once/day	260	47.7
1/day	130	23.9
2/day	99	18.2
3/day	56	10.3
FPMs with lesions due to caries		
No	350	64.2%
Yes	195	35.8%

Abbreviation: FPMs, first permanent molars.

Out of the total of 545 children, 280 (51.4%) were male and 265 (48.6%) were female, and no significant difference was found between FPMs with dental caries and gender (*p* > .05).

The proportion of FPMs with caries increased with the age of the children, and the prevalence of FPMs with caries was high in 11–12‐year‐old school children, with a statistically significant difference between FPMs with dental caries and age group (*p* < .05). A description of the clinical status of FPMs is summarized in Tables 2, 3, 4, 5.

**Table 2:** Status of the first upper right permanent molar (#16) by gender, age, and tooth brushing frequency

16								
	0: Sound	1: First visual change in the enamel	2: Distinct visual change in the enamel	3: Localized enamel breakdown	4: Underlying dark shadow from Dentin	5: Distinct cavity with visible Dentin	6: Extensive distinct cavity with visible Dentin	*p* Value
Gender								.743
Male	294 (45.7%)	5 (0.9%)	14 (2.6%)	4 (0.7%)	1 (0.2%)	1 (0.2%)	6 (1.1%)	
Female	237 (43.5%)	4 (0.7%)	8 (1.5%)	4 (0.7%)	1 (0.2%)	4 (0.7%)	7 (1.3%)	
Age group								.152
6–7	67 (12.3%)	0 (00%)	3 (0.6%)	0 (00%)	0 (00%)	0 (00%)	1 (0.2%)	
7–8	23 (4.2%)	0 (00%)	0 (00%)	1 (0.2%)	0 (00%)	0 (00%)	2 (0.4%)	
8–9	38 (7.0%)	0 (00%)	3 (0.6%)	3 (0.6%)	0 (00%)	0 (00%)	0 (00%)	
9–10	132 (24.2%)	2 (0.4%)	4 (0.7%)	2 (0.4%)	0 (00%)	1 (0.2%)	2 (0.4%)	
10–11	86 (15.8%)	2 (0.4%)	7 (1.3%)	1 (0.2%)	1 (0.2%)	2 (0.4%)	1 (0.2%)	
11–12	130 (23.9%)	5 (0.9%)	4 (0.7%)	1 (0.2%)	1 (0.2%)	2 (0.4%)	5 (0.9%)	
>12	10 (1.8%)	0 (00%)	1 (0.2%)	0 (00%)	0 (00%)	0 (00%)	2 (0.4%)	
Tooth brushing frequency								.473
Less than once/day	229 (42.0%)	7 (1.3%)	15 (2.8%)	3 (0.6%)	1 (0.2%)	1 (0.2%)	4 (0.7%)	
1/day	121 (22.2%)	0 (00%)	2 (0.4%)	2 (0.4%)	1 (0.2%)	1 (0.2%)	3 (0.6%)	
2/day	89 (16.3%)	1 (0.2%)	2 (0.4%)	1 (0.2%)	0 (00%)	2 (0.4%)	4 (0.7%)	
3/day	47 (8.6%)	1 (0.2%)	3 (0.6%)	2 (0.4%)	0 (00%)	1 (0.2%)	2 (0.4%)	

*p < .05.

**Table 3:** Status of the first upper left permanent molar (#26) by gender, age, and tooth brushing frequency

26								
	0: Sound	1: First visual change in the enamel	2: Distinct visual change in the enamel	3: Localized enamel breakdown	4: Underlying dark shadow from Dentin	5: Distinct cavity with visible Dentin	6: Extensive distinct cavity with visible Dentin	*p* Value
Gender								.398
Male	251 (46.1%)	3 (0.6%)	16 (2.9%)	4 (0.7%)	0 (00%)	0 (00%)	6 (1.1%)	
Female	234 (42.9%)	5 (0.9%)	13 (2.4%)	3 (0.6%)	3 (0.6%)	2 (0.4%)	5 (0.9%)	
Age group								.047*
6–7	69 (12.7%)	0 (00%)	1 (0.2%)	1 (0.2%)	0 (00%)	0 (00%)	0 (00%)	
7–8	24 (4.4%)	0 (00%)	0 (00%)	1 (0.2%)	0 (00%)	1 (0.2%)	0 (00%)	
8–9	40 (7.3%)	0 (00%)	3 (0.6%)	1 (0.2%)	0 (00%)	0 (00%)	0 (00%)	
9–10	125 (22.9%)	4 (0.7%)	11 (2.0%)	1 (0.2%)	0 (00%)	0 (00%)	2 (0.4%)	
10–11	89 (16.3%)	1 (0.2%)	5 (0.9%)	1 (0.2%)	1 (0.2%)	0 (00%)	3 (0.6%)	
11–12	130 (23.9%)	3 (0.6%)	6 (1.1%)	2 (0.4%)	2 (0.4%)	1 (0.2%)	4 (0.7%)	
>12	8 (1.5%)	0 (00%)	3 (0.6%)	1 (0.2%)	0 (00%)	0 (00%)	2 (0.4%)	
Tooth brushing frequency								.024*
Less than once/day	225 (41.3%)	7 (1.3%)	20 (3.7%)	3 (0.6%)	0 (00%)	1 (0.2%)	4 (0.7%)	
1/day	122 (22.4%)	0 (00%)	4 (0.7%)	0 (00%)	1 (0.2%)	0 (00%)	3 (0.6%)	
2/day	89 (16.3%)	0 (00%)	4 (0.7%)	1 (0.2%)	2 (0.4%)	0 (00%)	3 (0.6%)	
3/day	49 (9.0%)	1 (0.2%)	1 (0.2%)	3 (0.6%)	0 (00%)	1 (0.2%)	1 (0.2%)	

* *p* < .05.

**Table 4:** Status of the first lower left permanent molar (#36) by gender, age, and tooth brushing frequency

36								
	0: Sound	1: First visual change in the enamel	2: Distinct visual change in the enamel	3: Localized enamel breakdown	4: Underlying dark shadow from Dentin	5: Distinct cavity with visible Dentin	6: Extensive distinct cavity with visible Dentin	*p* Value
Gender								.643
Male	196 (36.0%)	9 (1.7%)	42 (7.7%)	15 (2.8%)	3 (0.6%)	10 (1.8%)	5 (0.9%)	
Female	174 (31.9%)	10 (1.8%)	47 (8.6%)	14 (2.6%)	2 (0.4%)	7 (2.0%)	11 (2.0%)	
Age group								.010*
6–7	63 (11.6%)	0 (00%)	7 (1.3%)	1 (0.2%)	0 (00%)	0 (00%)	0 (00%)	
7–8	23 (4.2%)	1 (0.2%)	0 (00%)	1 (0.2%)	0 (00%)	1 (0.2%)	0 (00%)	
8–9	29 (5.3%)	0 (00%)	6 (1.1%)	7 (1.3%)	0 (00%)	1 (0.2%)	1 (0.2%)	
9–10	96 (17.6%)	7 (1.3%)	24 (4.4%)	6 (1.1%)	1 (0.2%)	6 (1.1%)	3 (0.6%)	
10–11	60 (55.0%)	4 (0.7%)	25 (4.6%)	3 (0.6%)	3 (0.6%)	2 (0.4%)	3 (0.6%)	
11–12	94 (17.2%)	7 (1.3%)	24 (4.4%)	11 (2.0%)	1 (0.2%)	5 (0.9%)	6 (1.1%)	
>12	5 (0.9%)	0 (00%)	3 (0.6%)	0 (00%)	0 (00%)	3 (0.6%)	3 (0.6%)	
Tooth brushing frequency								.863
Less than once/day	173 (31.7%)	13 (2.4%)	41 (7.5%)	14 (2.6%)	1 (0.2%)	10 (1.8%)	8 (1.5%)	
1/day	9 (16.9%)	3 (0.6%)	21 (3.9%)	6 (1.1%)	2 (0.4%)	3 (0.6%)	3 (0.6%)	
2/day	65 (11.9%)	3 (0.6%)	19 (3.5%)	6 (1.1%)	2 (0.4%)	1 (0.2%)	4 (0.7%)	
3/day	40 (7.3%)	1 (0.2%)	8 (1.5%)	3 (0.6%)	0 (00%)	3 (0.6%)	1 (0.2%)	

* *p* < .05.

**Table 5:** Status of the first lower right permanent molar (#46) by gender, age, and tooth brushing frequency

46								
	0: Sound	1: First visual change in the enamel	2: Distinct visual change in the enamel	3: Localized enamel breakdown	4: Underlying dark shadow from Dentin	5: Distinct cavity with visible Dentin	6: Extensive distinct cavity with visible Dentin	*p* Value
Gender								.477
Male	198 (36.3%)	5 (0.9%)	46 (8.4%)	14 (2.6%)	2 (0.4%)	5 (0.9%)	10 (1.8%)	
Female	181 (33.2%)	13 (2.4%)	37 (6.8%)	16 (2.9%)	1 (0.2%)	5 (0.9%)	12 (2%)	
Age group								.001*
6–7	63 (11.6%)	0 (00%)	6 (1.1%)	1 (0.2%)	0 (00%)	0 (00%)	1 (0.2%)	
7–8	23 (4.2%)	0 (00%)	0 (00%)	2 (0.4%)	0 (00%)	0 (00%)	1 (0.2%)	
8–9	28 (5.1)	0 (00%)	7 (1.3%)	6 (1.1%)	0 (00%)	1 (0.2%)	2 (0.4%)	
9–10	100 (18.3%)	4 (0.7%)	22 (4%)	7 (1.3%)	0 (00%)	4 (0.7%)	6 (1.1%)	
10–11	63 (11.6%)	6 (1.1%)	18 (3.3%)	5 (0.9%)	2 (0.4%)	1 (0.2%)	5 (0.9%)	
11–12	97 (17.8%)	8 (1.5%)	27 (5.0%)	9 (1.7%)	2 (0.4%)	2 (0.4%)	4 (0.7%)	
>12	5 (0.9%)	0 (00%)	3 (0.6%)	0 (00%)	0 (00%)	2 (0.4%)	3 (0.6%)	
Tooth brushing frequency								.115
Less than once/day	173 (31.7%)	14 (2.6%)	42 (7.7%)	13 (2.4%)	0 (00%)	7 (1.3%)	11 (2.0%)	
1/day	103 (18.9%)	1 (0.2%)	15 (2.8%)	5 (0.9%)	1 (0.2%)	1 (0.2%)	4 (0.7%)	
2/day	63 (511.6%)	2 (0.4%)	18 (3.3%)	9 (1.7%)	2 (0.4%)	0 (00%)	5 (0.9%)	
3/day	40 (7.3%)		8 (1.5%)	3 (0.6%)	0 (00%)	2 (0.4%)	2 (0.4%)	

* *p* < .05.

In terms of brushing frequency and FPMs with dental caries, no statistically significant relationship was found, except for first upper left permanent molars, where a statistically significant difference was reported (*p* < .05).

In the present study, the prevalence of caries in the mandibular FPMs was significantly higher than the prevalence of caries in the maxillary FPMs (*p* < .05) (Table 6).

**Table 6:** D, M, and F components in FPMs

FPMs	Maxillary				Mandibular			
	16		26		36		46	
	*N*	%	*N*	%	*N*	%	*N*	%
Decayed	58	10.6	65	11.9	167	30.6	159	29.2
Missing	5	0.9	3	0.6	6	1.1	5	0.9
Filled	2	0.4	3	0.6	9	1.7	13	2.4
Total								
*p* value	*P* = .003*							

Abbreviation: FPMs, first permanent molars.

* *p* < .5.

The presence of surfaces affected by enamel defects was recorded in 4.3% of FPMs and presence of surfaces affected by fluorosis was recorded in 4.6% of FPMs, and no significant association was found between gingival bleeding, dental fluorosis, dental enamel defects (MIH), and caries activity in FPMs (Table 7).

**Table 7:** Distribution of participants with regard to gingival bleeding, malocclusion, dental fluorosis, and enamel defects according to FPMs with dental caries

FPMs	Lesions caused by caries		*p V*alue
	No *n* (%)	Yes *n* (%)	
Gingival bleeding			
Normal gingiva	350 (64.2)	165 (30.2)	.765
Mild inflammation	1 (0.2)	16 (2.9)	
Moderate inflammation	6 (1.1)	4 (0.7)	
Severe inflammation	1 (0.2)	2 (0.3)	
Malocclusion			
No	300 (55)	170 (31.2)	.950
Yes	50 (9.2)	25 (4.9)	
Dental enamel Defects			
Dental fluorosis			
Normal	339 (62.2)	181 (38.9)	.843
Questionable	3 (0.55)	6 (1.1)	
Very mild	6 (1.1)	2 (0.3)	
Mild	1 (0.2)	2 (0.3)	
Moderate	1 (0.2)	4 (0.7)	
Severe	00 (00)	00 (00)	
Molar hypoplasia			
Normal	340 (62.4)	182 (33.4)	.307
Pits hypoplasia	8 (1.4)	7 (1.2)	
Missing enamel	2 (0.3)	6 (1.1)	
Any other defects	00 (00)	00 (00)	
Extent of defect: <1/3	2 (0.3)	3 (0.5)	
Extent of defect: At least 1/3 < 2/3	7 (1.2)	8 (1.4)	
Extent of defect: At least 2/3	1 (0.2)	2 (0.3)	

Abbreviation: FPMs, first permanent molars.

## 4 DISCUSSION

Because FPMs play an important role in maintaining the dental and overall health of an individual, this study was mainly conducted on FPMs and aimed to determine the prevalence of dental caries in FPMs among school children aged between 6 and 13 years in a Tunisian rural community.

Despite the importance of these teeth, only a few articles studying the prevalence of FPMs with dental caries have been published in some countries. In Tunisia, only some studies have investigated the prevalence of dental caries though not exclusively on FPMs. To the best of our knowledge, this is the first report in Monastir, Tunisia, on the prevalence of caries in FPMs specifically.

Due to the differences in the design of studies, the age of the target groups, and other related variables, comparison of the present survey findings to previous global and national studies was difficult.

In the present study, 35.8% of the children (*n* = 195) had one or more FPMs with caries and 64.2% of the children (*n* = 350) did not have FPMs with caries.

This high prevalence is still considerably lower than that described in other studies.

A study conducted on 100 Moroccan children aged between 6 and 15 years showed that 77% of the children had FPMs with caries (Zouaidi et al., 2012). Another study conducted among 300 Sudanese children revealed that the prevalence of dental caries in FPMs was 61% (Abuaffan et al., 2018). In the kingdom of Saudi Arabia, a study conducted on 836 school children aged between 7 and 10 years showed that 66.4% of the examined children had FPMs with caries (Meer, 2012).

A second study conducted by Al‐Samadani and Ahmad 2012 in Jeddah, on 432 school children aged between 9 and 12 years, reported that only 24.5% of the children had intact FPMs.

In Taiwan, a study showed that among 333 first‐grade children, 48% of the children had caries‐free FPMs (Warren et al., 1997).

In contrast, in another city of the kingdom of Saudi Arabia, in a study conducted on 5394 FPMs, it was reported that only 16.5% of children had at least one decayed or filled FPMs and 83.5% of the children did not have caries (Alshiha et al., 2018).

In China, a few reports showed a wide variation in the prevalence of caries among children in the age range of 6–15 years. The prevalence of caries in the FPMs was reported in 8.7% of children in the age range of 7–8 years (Wang et al., 2012), 26.5% of children in the age range of 7–9 years (Riziwaguli et al., 2013), and 72% of children in the age range of 7–12 years (Su et al., 2012).

These results demonstrated a significant variation in the prevalence of caries worldwide; several factors such as socioeconomic level, dietary habits, cultures, and ethnicity could  be responsible for this variation.

The deep pits and fissures on the occlusal surface, the large‐sized crown, which leads to accumulation of acid produced by bacteria, early emergence of the tooth, improper oral hygiene of children, and the limited knowledge of parents may be considered as the main reasons for the high prevalence of caries in the FPMs.

In the present study, no significant difference was found between the prevalence of caries in FPMs and gender (*p* > .05), while the results obtained by Devaki among school‐going children in the age range of 6–14 years in Tenali Guntur showed that the prevalence of FPMs with caries was more frequent in boys than in girls (Devaki, 2011).

In terms of age, statistically significant differences were found between FPMs with dental caries and age groups (*p* < .05).

The proportion of FPMs with caries increased with the children's age, and the prevalence of FPMs with caries was higher in 11‐ to 12‐year‐old school children. Similar to our findings, in the literature, it has been found that with age, there is an increase in the prevalence of FPMs with caries among children (Al‐Samadani & Ahmad, 2012; Noronha et al., 1999; Su et al., 2012; Wang et al., 2012; Xue et al., 2015).

Almost half of the examined children (47.7%) reported brushing their teeth less than once a day, and no statistically significant relationship was found between brushing frequency and FPMs with dental caries, except for first upper left permanent molars, where a statistically significant difference was reported (*p* < .05). These results are not in agreement with those in the literature.

A systematic review conducted by Holmes 2016 showed that brushing less than twice a day led to a significant increase in lesions due to caries compared with ≥2/day brushing, and infrequent brushing was associated with an increase in lesions caused by caries. In this review, it was also found that individuals who reported brushing their teeth infrequently were at a higher risk of developing lesions due to caries than those who reported brushing more frequently, and this effect was more pronounced in the deciduous than in the permanent dentition, independent of the presence of fluoride in toothpaste.

In the present survey, the prevalence of caries in the mandibular FPMs was significantly higher than the prevalence of caries in the maxillary FPMs. This finding was in agreement with other studies (Al‐Samadani & Ahmad, 2012; Meer, 2012; Noronha et al., 1999; Wang et al., 2012; Xue et al., 2015).

This common finding may have been possibly due to the difference in the morphology and the earlier emergence of mandibular compared to maxillary FPMs. In fact, mandibular FPMs have more pits and supplementary grooves, which can act as food‐retentive areas. Some studies reported that in the majority of children, mandibular FPMs emerge slightly earlier than their maxillary counterparts, thus increasing the time of their exposure to the oral environment, making them more susceptible to caries (Meer, 2012).

A surprising result was found in the present study: indeed, even though 35.8% of the examined children had one or more FPMs with caries, only 5.1% of these FPMs were filled, and none of the children had a fissure sealant applied. Due to the lack of awareness among the public, the use of fissure sealants in Tunisian children is extremely low.

Similar to the findings of Alshiha et al. 2018, only in 0.8% of the children had fissure sealants been applied. The underuse of fissure sealants for prevention of caries among Tunisian children is alarming.

These disappointing results could be explained by the fact that children attending schools in rural communities visit the dentist only in the case of an emergency and cannot afford routine check‐ups or visits during which the dentist can perform preventative measures such as application of fluoride and fissure sealants, provide oral hygiene instructions, and guide the parents and children on proper oral hygiene maintenance and dietary control (Al‐Samadani & Ahmad, 2012).

Giving the excellent results obtained from other countries in terms of reduction of the prevalence of caries (Alshiha et al., 2018; Gooch et al., 2009; Klemme et al., 2004; Wendt et al., 2001), where school‐based fissure sealants programs were implemented, it is thus necessary to recommend introduction of the use of fissure sealants in school‐based or public preventive programs in Tunisia.

The presence of surfaces affected by severe molar incisor hypomineralization was recorded in 4.3% of children, and no significant association was found between molar incisor hypomineralization and the prevalence of caries in FPMs. These results were not in agreement with those reported in a systematic review that identified a positive association between enamel defects and dental caries (Vargas‐Ferreira et al., 2015).

An early preventive program like application of fissure sealants and meticulous home care could help to reduce the prevalence of caries in FPMs (Al‐Samadani & Ahmad, 2012; Mimoza & Vito, 2018).

Based on the present results, school‐going children in Tunisia should have periodic dental visits, be encouraged to brush their teeth daily, and follow a low‐carbohydrate diet. Also, it is very essential to focus on systematic oral health promotion measures in school programs.

The present study had certain limitations such as the sample size, which was not large enough to enable generalization of the results. Because the study was carried out as part of an annual dental preventive program organized by the Preventive Dentistry Department and because just one questionnaire intended for children was used, only tooth brushing frequency as an important determinant of dental caries was studied.

## 5 CONCLUSION

The high prevalence of caries in FPMs among schoolchildren shows that dental caries is still a major health problem; therefore, educating parents and teachers on the importance of FPMs and promoting children's oral health care are still necessary.

It is also very important to determine the frequency and severity of caries affecting FPMs to establish programs to prevent their decay because, from functional and developmental points of view, FPMs are the most important teeth in the dental arch.

## AUTHOR CONTRIBUTIONS

Farah Chouchene and Fatma Masmoudi conceived the idea, collected the data, and wrote the article. Farah Chouchene, Fatma Masmoudi, and Ahlem Baaziz analyzed the data. Hichem Ghedira provided comprehensive judgment and assisted with editing of the final version of the manuscript. All authors read and approved the final version of the manuscript before submission.

## CONFLICT OF INTEREST

The authors declare no conflict of interest.

## Data Availability

Data are available on request due to ethical restrictions.
